# What’s Race got to do With it? How Palliative Care Consultation may Mitigate Racial Disparities in Future Care

**DOI:** 10.1093/geroni/igab046.3174

**Published:** 2021-12-17

**Authors:** Lauren Starr, Connie Ulrich, G Adriana Perez, Subhash Aryal, Paul Junker, Nina O'Connor, Salimah Meghani

**Affiliations:** 1 University of Pennsylvania, Philadelphia, Pennsylvania, United States; 2 University of Pennsylvania School of Nursing, Philadelphia, Pennsylvania, United States; 3 University of Pennsylvania School of Nursing, Phildelphia, Pennsylvania, United States; 4 University of Pennsylvania Health System, Philadelphia, Pennsylvania, United States; 5 Penn Medicine, Perelman School of Medicine, Philadelphia, Pennsylvania, United States

## Abstract

It is unknown if care and cost outcomes differ by race and ethnicity following discharge from a hospitalization involving palliative care consultation to discuss goals-of-care (PCC). In this secondary analysis of 1,390 seriously-ill patients age 18+ alive at discharge who self-identified as Black, Hispanic, Asian, white, or other race and received PCC at an urban, academic medical center, we used binomial logistic regression and multiple linear regression controlling for demographic and clinical variables to identify factors associated with care experiences and costs following discharge from a hospitalization with PCC. In adjusted analyses, discharge to hospice was associated with Medicaid (p=0.016). Thirty-day readmission was associated with age 75+ (P=0.015), Medicaid (P=0.004), admission 30 days prior (P<0.0001), and Black race compared to white (P=0.016). Number of future days hospitalized was associated with Medicaid (P=0.001), admission 30 days prior (P=0.017), and Black race compared to white (P=0.012). Having any future hospitalization cost was associated with patient ages 65-74 (P=0.022) and 75+ (P=0.023), Medicaid (P=0.014), admission 30-days prior (P<0.0001), and Black race compared to white (P=0.021). Total future hospitalization costs were associated with female gender (P=0.025), Medicaid (P=0.009), admission 30 days prior (P=0.040), and Black race compared to white (P=0.037). Race or ethnicity was not a predictor of hospice enrollment. Randomized controlled trials are needed to understand if PCC is an intervention that reduces racial disparities in end-of-life care. Qualitative insights are needed to explain how PCC and socioeconomic factors such as Medicaid may mitigate future acute care use among racial and ethnic groups.

